# Supplementary figures and images for: Axial light loss of monocytes as a readily available prognostic biomarker in patients with suspected infection at the emergency department
Source: PLoS One. 2022 Jul 11;17(7):e0270858. doi: 10.1371/journal.pone.0270858 (PMC9273078; doi:10.1371/journal.pone.0270858)

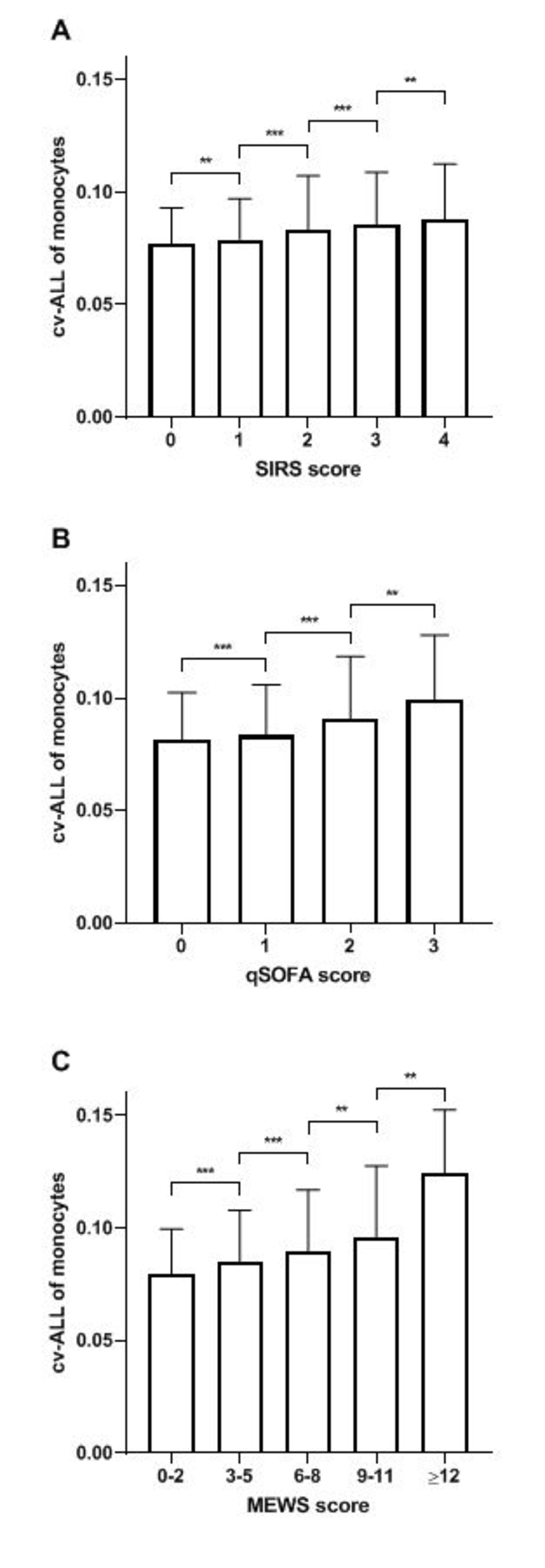

Supplement: S1 Fig — SIRS (A), qSOFA (B), and MEWS (C) score and height of cv-ALL of monocytes is shown. A one-way ANOVA was performed to test group differences. Significance testing was done by Tukey’s test. **p < 0.01, ***p < 0.001. (TIF) [file pone.0270858.s001.tif]

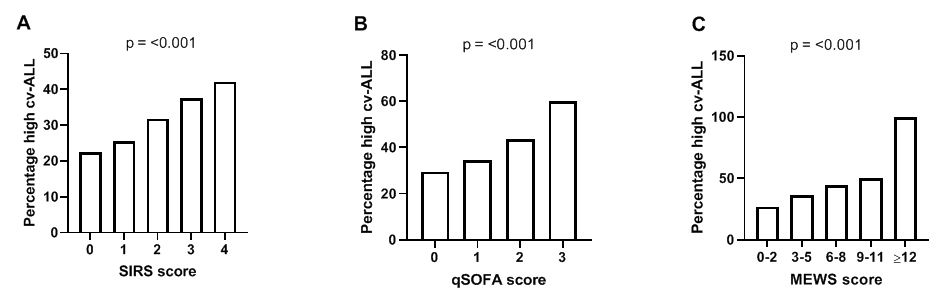

Supplement: S2 Fig — High cv-ALL of monocytes percentage for SIRS (A), qSOFA (B), and MEWS (C) scores. High cv-ALL of monocytes was defined as the cut-off value for our clinical model to predict 30-day mortality (0.085). P-values were calculated with a X-square test. (TIF) [file pone.0270858.s002.tif]

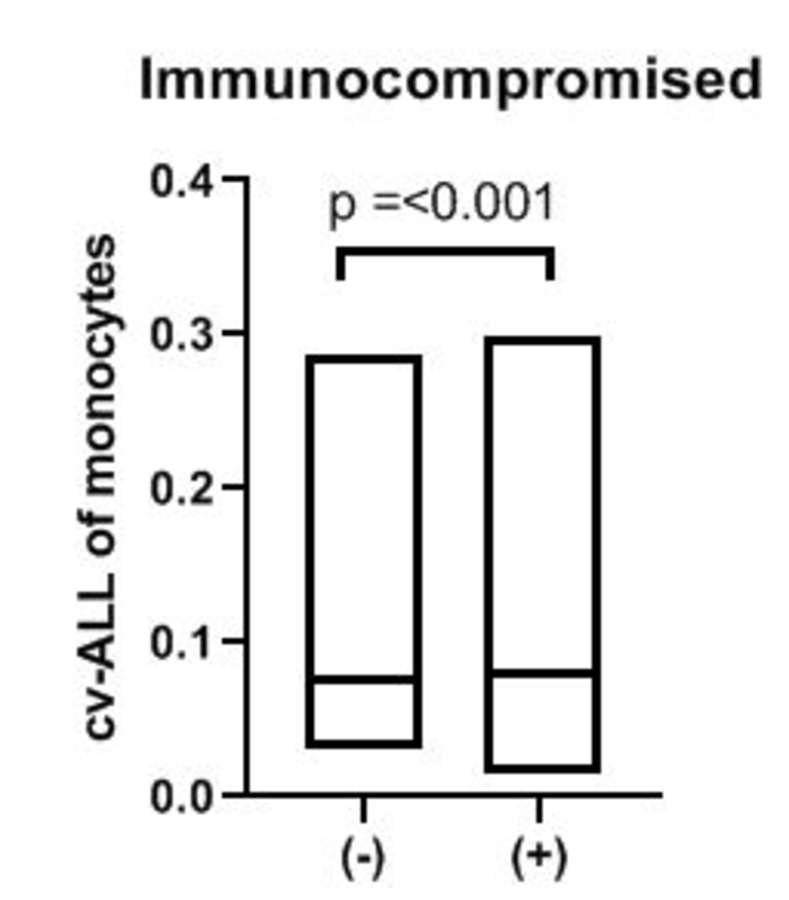

Supplement: S3 Fig — Cv-ALL of monocytes differed significantly between these two groups. P-value was calculated by a Mann-Whitney U test. (TIF) [file pone.0270858.s003.tif]

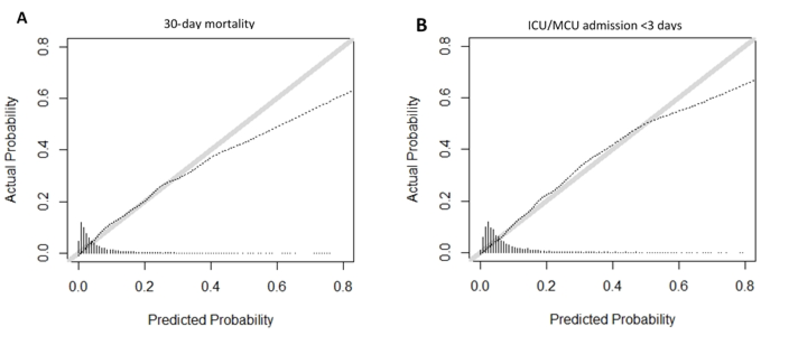

Supplement: S4 Fig — Optimal model calibration plots for 30-day mortality (A) and ICU/MCU admission <3 days (B). The dashed line shows the calibration plot for the optimal models. The model for 30-day mortality has R2 of 0.209 and Brier score of 0.054, while the model for ICU/MCU admission <3 days shows R2 of 0.220 and Brier score of 0.070. (TIF) [file pone.0270858.s004.tif]
