# Supplementary material for: Axial light loss of monocytes as a readily available prognostic biomarker in patients with suspected infection at the emergency department
Source: PLoS One. 2022 Jul 11;17(7):e0270858. doi: 10.1371/journal.pone.0270858 (PMC9273078; doi:10.1371/journal.pone.0270858)
Supplement: Attachment — Submitted filename: Response to reviewers.docx [file pone.0270858.s005.docx]

# **Response to reviewers**

Comments of the Editor

1. Can you explain what "semi-automated" data collection is? how much was done by computer vs hand and was there any manual checking of data pulled automatically?

Response

We agree that semi-automated is a vague term. Therefore, we explained what type of data was collected manually and what type of data was collected via automated queries.

- Line 130: For all included patients, data concerning demographics, vital parameters, antibiotics, comorbidities, and outcome was collected manually and supplemented with automated queries for laboratory variables to calculate cv-ALL of monocytes. Data on vital parameters included all parameters to calculate clinical prediction scores (SIRS, qSOFA, MEWS) and follow-up data on morbidity and mortality included microbiological diagnostics, chosen treatment, hospitalization, and length of stay).

2. Please include a detailed explanation of missing data

Response

Thank you for addressing this. Fortunately, we had not much missing data. To be completely transparent on this issue, we added the following text:

- Line 159: Concerning data points on laboratory variables, hospital admission, and clinical course, all used parameters had missing data <1%. This was also the case for all used vital parameters, except for respiratory rate (missing 25.8%). No data on demographics were missing.

3. Please describe the multiple imputation process in more detail

Response

Thank you for adding this transparency to the manuscript. The multiple imputation process was described in more detail:

- Line 157: We used a predictive mean matching multiple imputation approach for missing values. All included vital, laboratory and outcome parameters that were used in our analyses were used.

4. Please include details about model calibration

Response

We understand the interest in calibration of our model and therefore included Hosmer-Lemeshow analyses to our manuscript. However, the main purpose of this manuscript was to address the added value of cv-ALL of monocytes to current clinical prediction scores instead of producing a clinical model that could be implemented in clinical practice. Therefore, the clinical models are not essential for our main message.

5. The discussion is well-written overall and makes some very good points. however, i think that the tone is a bit too conclusive in that this is a single retrospective study from a single center. In addition, automated variable selection techniques are notorious for creating over-fit models--while i think this is a reasonable first step, i do not think these data can be considered definitive. furthermore, prospective validation is definitely needed before this should be used in routine clinical practice. i would recommend that the discussion be modified slightly to reflect this fact.

Response

We thank the editor for this suggestion and agree on this point. Therefore, the discussion was rewritten in a less conclusively and the following section was added:

- Line 286:At last, we show that multivariate models can achieve good AUCs to predict outcome. However, these models showed a poor goodness of fit and were not validated. Therefore, these models are not suitable for implementation in clinical practice yet.

Comments of the reviewer

1. It is a little unclear whether or not all the patients in the study timeframe were included if they were >=18 and had a suspected infection or whether or not it required an abnormal SIRS or qSOFA score.

Response

Thank you for pointing this out. Indeed all patients were included. This was stated in paragraph 2.1 (line 105-109). Also, in Table 1 we describe the amount of patients with abnormal SIRS/qSOFA/MEWS scores, implying that patients with normal scores were included as well.

2. This was a post-hoc analysis of a prospectively collected database and blood samples. The cv-ALL contributed to improving the performance of know disease severity scores. That said the baseline characteristics of the survivors vs. non-survivors were not balanced. The OR of qSOFA and MEWS was much more robust for the selected outcomes that cv-ALL alone.

Response

It is correct that baseline characteristics were not balanced. We do not fully

understand what the reviewer means by differing robustness of the ORs. To our opinion the confidence intervals give information about robustness of the results and these are not broader for cv-ALL than for the disease severity scores.

3. Was lactate included in the analysis? cv-ALL is independent of overall WBC? It would suggest yes – given that they are both in the final model, however one would think there is significant interaction b/w these variables.

Response

Thank you for this critical note. Unfortunately, lactate could not be included in the analyses because we only have lactate in a small fraction of our population. Concerning the independence of cv-ALL and WBC, we tested this with a Pearson correlation test which resulted in a correlation coefficient of -0.081. Due to this negligible correlation coefficient, we are convinced that cv-ALL is independent of WBC. We hypothesize that it is unlikely that the absolute amount of leukocytes in the peripheral blood influences the variation in monocyte size.

4. What is the rationale for a higher CCI being less likely to be admitted to the MCU/ICU – is it related to ICU-restrictions of these patients?

Response

Although ICU-restrictions would seem to be a logical explanation for this difference, we already excluded all patients with ICU-restrictions for these analyses. This was also stated in line 150-151. Therefore, we do not know what causes this difference exactly. It is possible that even if patients do not have treatment restrictions, clinicians are less likely to treat patients with high CCI scores at the ICU due to low chances for a good outcome.

5. I would have liked to see some basic data concerning process measures such as time to antibiotics which is strongly correlated with outcome in the sicker sepsis/septic shock patients.

Response

Thank you for this suggestion and we agree this would be interesting information for our manuscript. Therefore, we added “timing of antibiotics” and “length of stay” and “hospital admission” to our baseline table (Table 1). As can be seen, antibiotics were more frequently prescribed in the non-surviving group and more often were these antibiotics given directly (<1 hour) or early (1-3 hours) when compared to survivors.
