# Supplementary material for: Axial light loss of monocytes as a readily available prognostic biomarker in patients with suspected infection at the emergency department
Source: PLoS One. 2022 Jul 11;17(7):e0270858. doi: 10.1371/journal.pone.0270858 (PMC9273078; doi:10.1371/journal.pone.0270858)
Supplement: Attachment — Submitted filename: Response to reviewers.docx [file pone.0270858.s006.docx]

# **Response to reviewers**

Comments of the Editor

My remaining concern relates to non-reporting of model calibration in the current iteration of the paper (NB: HL test is a goodness of fit measure rather than one of calibration). To me, regardless of how (or even if) one intends prediction models to be used, calibration is an important data point to include--calibration and discrimination (eg, AUC) go hand-in-hand.

We thank the editor for this suggestion and we agree the manuscript would benefit from a calibration. Therefore, we included a calibration plot (including R^2^ and Brier score) in the manuscript. Details of the calibration are described in the methods (line 172-174) and results (lines 200-201 + 243-244) section. Also, implications are discussed in the discussion (lines 288-291) and calibration plots were included as supplemental figure S4.
